# 

**Published:** 1898-05-28

**Authors:** 


### NOTICE TO CORRESPONDENTS.

All MS., Letters, Books for Review, and other matters intended for the Editor should be addressed THE EDITOR, "THE HOSPITAL," THE HOSPITAL BUILDING, 28 & 29, SOUTHAMPTON STREET, STRAND, W.C.

The Editor cannot undertake to return rejected MS., even when accompanied by stamped directed envelope.

**Notice to Advertisers and Subscribers.**

All Advertisements, Orders for Copies of THE HOSPITAL, and Business Communications should be addressed to **THE MANAGER, (Not to The Editor),** THE HOSPITAL, The Hospital Building, 28 & 29, Southampton Street, Strand, London, W.C.

The Cost of Subscription, post free, is as follows:—Per week, 2½d. per quarter, 2s. 8d.; per half-year, 5s. 3d.; per annum, 10s. 6d. Foreign:— Per annum, 15s. 2d.; payable, in all cases, in advance.

### The Student of Medicine.

### APPOINTMENTS.

C. F. M. Althorp, M.B.Lond., M.R.C.S., L.R.C.P.Lond., appointed Honorary Surgeon to the Bradford Royal Infirmary. J. F. Atkins, M.R.C.S.Eng., L.R.C.P.Lond., appointed Medical Officer to Messrs. Tangye's Cornwall Works, Smethwick, Birmingham. C. K. Bowes, M.A., M.B., B.Ch.Oxon., appointed Medical Officer for the St. Anne's Home, Herne Bay. A. H. Burgess, C.L.B.Vict., appointed Medical Officer for the Crumpsall Workhouse, Manchester. F. P. Cayley, M.R.C.S.Eng., L.R.C.P.Lond., appointed Clinical Assistant at the Whitechapel Union Infirmary. K. C. Chetwood-Aiken, appointed Ophthalmic Surgeon to the Royal Cornwall Infirmary. W. H. Cooper, M.R.C.S., L.R.C.P., appointed Medical Officer for the Staveley District of the Kendal Union. C. V. Dingle, M.D., B.Sc.Durh., B.Hy., appointed Medical Officer of Health for Middlesbrough. A. Ehrmann, M.R.C.S., L.R.C P., appointed Clinical Assistant to the Chelsea Hospital for Women. Dr. D. Fraser, appointed Medical Officer of Health to the Buckley Urban District Council. G. Hardwick, M.R.C.S., L.R.C.P., appointed Resident Medical Officer at Ida Hospital. Mr. E. E. Lloyd, appointed Junior Assistant Medical Officer to the Fulham Union Infirmary. Dr. Mackay, appointed Medical Officer of Health to the Crook Urban District Council. Dr. Macpherson, appointed Medical Officer and Public Vaccinator for the Bere Ferrers District of the Tavistock Union. H. Markley, M.R.C.S., L.R.C.P., appointed House Surgeon to the General Infirmary, Leeds. A. G. Martineau, M.R.C.S., L.R.C.P., appointed House Surgeon to the Nottingham General Hospital. W. Overend, M.D., B.Ch., appointed a Clinical Assistant to the Chelsea Hospital for Women. C. Pierson, M.R.C.S., L.R.C.P., appointed House Physician to the General Infirmary, Leeds. I. G. Sibbald, M.B., C.M.Edin., appointed House Surgeon to the Northern Infirmary, Inverness. G. B. Simpson, L.R.C.P., L.R.C.S.Edin., appointed Medical Officer for the Lastingham District of the Pickering Union. F. Talbot, M.R.C.S., L.R.C.P., appointed House Surgeon to the General Infirmary, Leeds. A. F. Turner, L.R.C.P., L.R.C.S.Edin., appointed Medical Officer of Health for the Borough of Tewkesbury. H. Veale, L.S.A., appointed House Physician to the General Infirmary, Leeds.

